# Adaptive lift chiller units fault diagnosis model based on machine learning

**DOI:** 10.1371/journal.pone.0320563

**Published:** 2025-04-24

**Authors:** Yang Guo, Zengrui Tian, Hong Wang, Mengyao Chen, Pan Chu, Yingjie Sheng

**Affiliations:** 1 College of Building Environment Engineering, Zhengzhou University of Light Industry, Zhengzhou, China; 2 Henan Engineering Research Center of Intelligent Buildings and Human Settlements, Zhengzhou, China; Aalto University, FINLAND

## Abstract

The early minor faults generated by the chiller in operation are not easy to perceive, and the severity will gradually increase with time. The traditional fault diagnosis method has low accuracy and poor stability for early fault diagnosis. In this paper, a fault diagnosis model of Chiller is designed by combining least squares support vector machine (LSSVM) optimized by hybrid improved northern goshawk optimization algorithm (HINGO) and improved IAdaBoost ensemble learning algorithm. HINGO enhances the uniformity of the initial population distribution by means of refraction opposition-based learning strategy in initialization, and improves the local and global search ability of the algorithm by means of sine and cosine strategy, Lévy flight and nonlinear decreasing factor in the search stage. The HINGO-LSSVM-IAdaBoost model is trained and validated on the typical air conditioning fault samples of ASHRAE RP-1043. Compared with the traditional methods, the HINGO-LSSVM-IAdaBoost model shows obvious advantages for the early fault diagnosis of chiller units.

## Introduction

Energy consumption in the building sector accounts for approximately 20%–40% of global total energy consumption. Among all energy-consuming devices within buildings, heating, ventilation, and air conditioning (HVAC) systems account for over 50% of total energy consumption [1]. As a core component of HVAC system, chiller unit account for 50–60 percent of the total energy consumption of building operations [2]. Due to its complex structure and working environment, chiller units often face various types of failures, which not only reduce the system efficiency and energy efficiency, but also shorten the service life of the equipment [3]. 30% of the air conditioning and refrigeration system energy waste is caused by chiller unit failures [4], and the energy waste of the chiller can be significantly reduced by using fault diagnosis technology to improve the accuracy of fault removal. Therefore, the study of condition monitoring and fault diagnosis of chillers is of great significance.

Fault Detection and Diagnosis (FDD) technology can be divided into three categories: Physics-based methods rely on physical laws and historical data to establish mathematical models [5]. Expert knowledge-based methods combine expert experience to build knowledge bases, suitable for systems that are difficult to model but requiring significant prior knowledge and expert involvement [6]. Data-driven methods collect large datasets from actual systems and apply machine learning for fault detection, suitable for high-dimensional and complex nonlinear problems. With the development of technology, data-driven methods have been widely applied in various fault diagnosis fields [7].

In the field of bearing fault diagnosis. Literature [8] proposed a train bearing fault diagnosis method based on improved time-shifted multi-scale fractional-order fuzzy dispersion entropy and hybrid kernel ridge regression (RTSMFFDE-HKRR) for noisy environments. RTSMFFDE combines refinement, time-shifting, and fractional-order theory to enhance the stability of entropy. A hybrid kernel function combining radial basis function (RBF) and linear kernel is constructed. Experimental results show that RTSMFFDE can effectively extract comprehensive fault feature information under noisy conditions, significantly improving the classification performance of HKRR and making it suitable for train bearing fault diagnosis in noisy environments.

With the increasing complexity of building energy systems and the gradual advancement of artificial intelligence and building automation technologies, data-driven approaches have been widely applied in building performance simulation [9], system optimi zation [10], and FDD of HVAC systems including vapor compression refrigeration (VCR) systems [11], variable refrigerant flow (VRF) systems [12], air handling units (AHUs) [13], building ventilation fans [14], and chiller unit [15].

Commonly used data-driven methods include decision trees, support vector machines (SVM), neural networks, principal component analysis (PCA), and independent component analysis (ICA). Currently, support vector machine (SVM) have been widely used for fault diagnosis in HVAC systems.

SVM maps the training data into a high-dimensional space by introducing a nonlinear feature function, maximizing the separation margins of two different classes in the feature space while minimizing the training error using conventional optimization methods [16]. Literature [17] developed a multilayer SVM classifier to identify faults in single zone HVAC systems. Literature [18] investigated a hybrid FDD method for chillers that combines an autoregressive model with exogenous variables and SVM and compared it with a multilayer perceptron neural network classifier, and the results showed the advantages of the proposed method in terms of improved prediction accuracy and reduced false alarm rate. Although SVM has high generalisation ability and can effectively solve the problem of fault classification. The complexity of the constrained quadratic programming problem in SVM is closely tied to the sample size. Large datasets can significantly slow down training and consume excessive memory. This leads to increased computational complexity and reduced efficiency [19].

To solve the problem of the high computational complexity of SVM, Suykens and Vandewalle [20] proposed a Least Squares Support Vector Machine (LSSVM), the use of equational constraints instead of inequality constraints in SVM effectively reduces the computation and solves the problem of high computational complexity and difficult training of support vector machines on large-scale datasets [21]. Literature [22] proposed a fault diagnosis model based on Extended LSSVM (E-LSSVM), offering higher generalization ability and lower computational complexity than traditional SVM. This provides a more effective solution for fault diagnosis. The penalty factor *C* and kernel parameter *g* of LSSVM greatly affect the model’s accuracy. Traditional parameter settings are heavily influenced by subjective factors. Inappropriate parameter settings can lead to a decline in model performance. Literature [23] proposed a fault diagnosis method for LSSVM optimised by Gravitational Search Algorithm (GSA) and used fault samples from ASHRAE RP-1043 chiller operation reports. The experimental results show that the model’s overall average fault diagnosis accuracy for early failures in the chilled water units exceeds 95%. However, LSSVM is sensitive to outliers due to its training method, which minimizes the squared loss function, leading to significant errors when facing noise or anomalous data.

AdaBoost is an ensemble learning method that enhances robustness against noisy data by focusing on previously misclassified samples. After each iteration, AdaBoost increases the weights of misclassified samples, making the model pay more attention to difficult-to-classify instances, thereby improving the overall diagnostic accuracy of the model. Its function is similar to human learning processes, where each learner builds upon the knowledge of its predecessor and corrects the mistakes made by earlier learners. The strong classifiers generated through the AdaBoost approach preserve the strengths of individual weak classifiers while mitigating their limitations [24]. Literature [25] proposed an improved Generative Adversarial Network (IGAN) combined with an Enhanced Deep Extreme Learning Machine (EDELM) method. This method integrates the Multi-Head Attention (MHA) mechanism into the traditional Generative Adversarial Network (GAN) to generate new samples that conform to the distribution of minority class fault samples and rebalance the dataset. It uses the Deep Extreme Learning Machine (DELM) as the base classifier. An Adaptive Boosting (AdaBoost) strategy is employed. This strategy integrates multiple DELM base classifiers through a weighted voting approach to form the final ensemble classifier. Experimental results show that this method achieves high fault diagnosis accuracy in imbalanced data environments.

Although there have been studies on chiller fault diagnosis, there are fewer studies on the diagnosis of faults in the early stages of operation. In the daily operation of a chiller, when the severity of a fault is low, its impact on system operation is minimal, making initial faults difficult to identify. If minor faults are ignored, they can lead to an increase in severity and cause catastrophic damage to the system. Timely identification of initial faults can help reduce equipment downtime, energy wastage, and maintenance costs [26]. Traditional diagnostic methods may struggle with diagnosing early-stage faults in water chillers, often showing lower accuracy and stability. This paper introduces an adaptive enhanced fault diagnosis method based on the HINGO-LSSVM-IAdaBoost approach. The feasibility and advantages of this method are confirmed using the ASHRAE RP-1043 air conditioning chiller unit experimental dataset. Our results suggest that this proposed method greatly improves the diagnostic accuracy for initial faults during the operation of air conditioning chillers.

(1) To address the issue of LSSVM model performance being significantly influenced by the penalty factor *C* and kernel parameter *g*, this study selects the Northern Goshawk Optimization (NGO) algorithm for parameter optimization. To overcome the inherent limitations of the Northern Goshawk Optimization algorithm, a Hybrid Improved Northern Goshawk Optimization algorithm (HINGO) is proposed. This algorithm integrates the Refraction Backward Learning (ROBL) method, the Sine Cosine Algorithm, nonlinear weighting factors, and the *Lévy* flight strategy to enhance the algorithm’s search capability.
(2) Based on the traditional AdaBoost algorithm, this paper proposes an improved weighted update rule called IAdaBoost (Improved AdaBoost). This algorithm addresses the issue of rapid weight escalation due to misclassified samples by allowing for flexible adjustment of classifier weights. Additionally, the HINGO algorithm is utilized to optimize the parameters of the LSSVM, constructing the HINGO-LSSVM fault diagnosis model as a weak classifier. The model is iteratively trained using the IAdaBoost algorithm, with weights adjusted according to the accuracy of each weak classifier. Ultimately, the weighted voting results from each weak classifier are aggregated to form a robust strong classifier, establishing the HINGO-LSSVM-IAdaBoost adaptive fault diagnosis model.
(3) The HINGO-LSSVM-IAdaBoost fault diagnosis method is validated using the ASHRAE RP-1043 dataset, along with ablation experiments. Various evaluation metrics are used to assess the model’s diagnostic performance. Experimental results show that the proposed method achieves high diagnostic accuracy on the RP-1043 dataset and can accurately detect early faults in air conditioning systems. The ablation experiment results demonstrate the importance of each improvement module in enhancing fault diagnosis accuracy and robustness.

## Introduction of chiller unit

Chiller unit is the main component of HVAC system, has been widely used in air conditioning and industrial refrigeration field. By accurately controlling the temperature and flow of cooling water and frozen water, the chiller can effectively regulate the indoor temperature and maintain the stability of indoor humidity, so as to provide users with a comfortable and healthy environment

**Fig 1:**
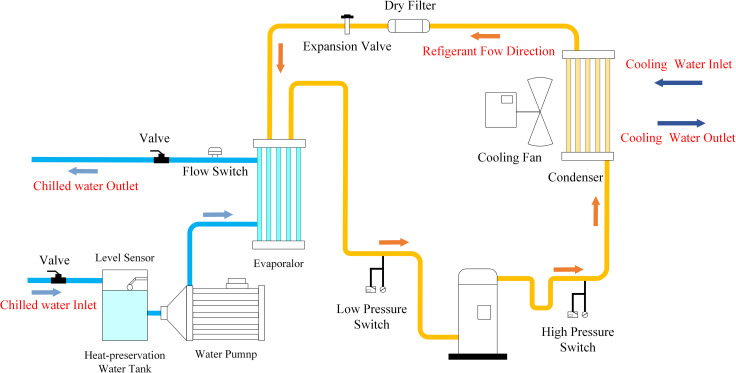
Schematic diagram of chiller units [25].

The American Society of Heating, Refrigerating, and Air-Conditioning Engineers (ASHRAE) conducted a simulated failure experiment on centrifugal chillers in 1999 and released the ASHRAE RP-1043 database [27]. Since the release of the ASHRAE data, many scholars have applied the database in their research on various approaches to chiller FDD. The chiller unit system is shown in Fig 1.

Operational data from a water-cooled chiller unit documented in the ASHRAE Research Project RP-1043 report includes seven typical faults, as detailed in Table 1. For these seven typical faults, the RP-1043 dataset categorizes each fault into four distinct severity levels, ranging from SL1 (least severe) to SL4 (most severe). To validate the fault diagnosis model’s accuracy in detecting minor early faults, this study focuses on utilizing monitoring data corresponding to SL1 faults along with a set of normal operational data to form a sample dataset. The experimental dataset comprises 11,000 samples selected from eight types of data, divided into training and testing sets at a ratio of 7:3.

**Table 1:** Description of fault types in the RP-1043 dataset.

Fault number	Fault type	Abbreviation
1	Excessive over	EO
2	Condenser fouling	CF
3	Refrigerant leak	RL
4	Refrigerant Over	RO
5	Non-condensable gas contained	NC
6	Flow water of condenser insufficient	FWC
7	Flow water of evaporator insufficient	FWE

The RP-1043 project collected data from 64 variables of the chiller unit, but most features showed insufficient sensitivity to faults. Based on the research in literature [28], it was verified that 16 of these features are suitable as input variables for fault diagnosis of the chiller unit. These 16 features have been used in fault diagnosis experiments by several scholars, such as in literature [29] and [30]. This paper will use these 16 variables for further study.

## Chiller units fault diagnosis model based on HINGO-LSSVM-IAdaBoost

### Least squares support vector machines

The set of training samples is set to D={(*x*_*1*_*,y*_*1*_),...,(*x*_*n*_*,y*_n_)}, *x*_n_∈*R*_*m*_, represents the feature vector of the sample, *m* represents the sample dimension, *n* represents the number of samples, *y*_*n*_ ∈{+1, -1}. The objective function of the optimization problem is as follows:


$$\{\begin{array}{l} \min J(\omega ,b,e)=\frac{1}{2}\omega^{T}\omega +\frac{1}{2}C\sum_{i=1}^{N}e_{i}^{2},\gamma >0\\ s.t.y_{i}[\omega^{T}\varphi (x_{i})+b]=1-e_{i},i=1,...,N \end{array}$$ (1)

In the formula *φ* is a nonlinear mapping, *ω* is a hyperplane weight vector; *b* is the bias factor; *C* is the coefficient of penalty term; *e*_*i*_ is the slack variable. By introducing the Lagrangian operator *α*_*i*_, Eq. (1) is rewritten as follows.


$$L(\omega ,b,e,\alpha )=J(\omega ,b,e)-\sum_{i=1}^{N}\alpha_{i}\left\{y_{i}[\omega^{T}\varphi (x_{i})+b]-1+e_{i}\right\}$$ (2)

The optimal solution problem can be transformed into:


$$\frac{\partial \text{L}}{\partial \omega }=0\to \omega ==\sum_{i=1}^{N}\alpha_{i}y_{i}\varphi (x_{i})$$ (3)

$$\frac{\partial L}{\partial b}=0\to \sum_{i=1}^{N}\alpha_{i}y_{i}=0$$ (4)

$$\frac{\partial L}{\partial e_{i}}=0\to \alpha_{i}=ye_{i}$$ (5)

$$\frac{\partial L}{\partial \alpha_{i}}=0\to y_{i}[\omega^{T}\varphi (x_{i})+b]-1+e_{i}=0$$ (6)

The matrix form of Eqs. (3)–(6) is as follows.


$$\left[\begin{array}{cc} 0 & E^{T}\\ E & \Omega +E_{\gamma }^{-1} \end{array}\right]\left[\begin{array}{c} b\\ \alpha \end{array}\right]=\left[\begin{array}{c} 0\\ y \end{array}\right]$$ (7)

$$\Omega_{ij}=\varphi {\left(x_{i}\right)}^{T}\varphi (x_{j})=K(x_{i},x_{j})\text{i,j=1,2,}...\text{,N}$$ (8)

In Eq. (8), *K*(*x*_*i*_*, x*_*j*_) denotes the kernel function. It can reduce the computational complexity when building a high-performance least squares support vector machine. The radial basis function is chosen as the kernel function in this paper. After obtaining *α* and *b*, the decision function for classification can be obtained as follows.


$$y(x)=sign\left[\sum_{i=1}^{N}\alpha_{i}y_{i}K(x,x_{i})+b\right]$$ (9)

**Fig 2:**
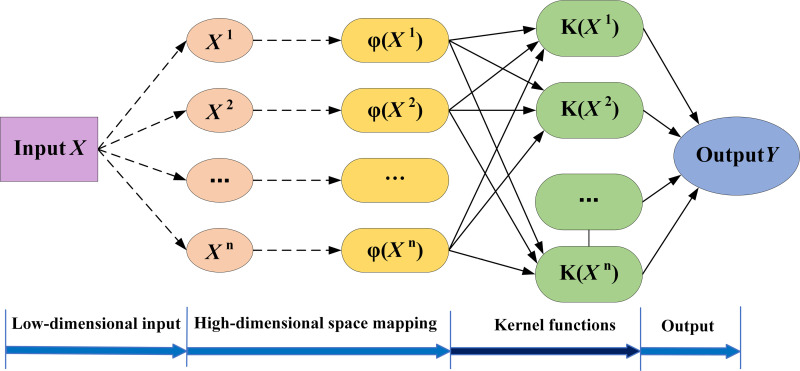
Least squares support vector machine model.

LSSVM is sensitive to regularization coefficients and kernel parameters. Therefore, it needs to be optimized appropriately. The LSSVM model is shown in Fig 2.

### Northern Goshawk optimization

Northern goshawk optimization (NGO) includes two stages: prey identification and attack (exploration stage), and chase and escape (development stage) [31].

(1) Initialization phase. The Northern Goshawk is randomly initialized in the search space, as shown in Eq. (10).

$$X={\left[\begin{array}{c} x_{1}\\ x_{2}\\ \vdots \\ x_{n} \end{array}\right]}_{N\times \text{m}}={\left[\begin{array}{ccccc} x_{1,1} & \cdots & x_{1,j} & \cdots & x_{1,m}\\ \vdots & \ddots & \vdots & \ddots & \vdots \\ x_{i,1} & \cdots & x_{i,j} & \cdots & x_{i,m}\\ \vdots & \ddots & \vdots & \ddots & \vdots \\ x_{N,1} & \cdots & x_{N,j} & \cdots & x_{N,m} \end{array}\right]}_{N\times \text{m}}$$ (10)

*X*_*i*_ is the initial solution of the ith individual, *x*_*ij*_ is the value of the *j*th dimension of the *i*th individual, *N* is the number of individuals, and *m* is the spatial dimension. Eq. (11) is the objective function value:


$$F(X)={\left[\begin{array}{c} F_{1}=F(X_{1})\\ \vdots \\ F_{i}=F(X_{i})\\ \vdots \\ F_{N}=F(X_{N}) \end{array}\right]}_{N\times 1}$$ (11)

where *F* is the vector of objective function values and *F*_*i*_ is the objective function value of the *i*th solution.

(2) The first stage: exploration stage. In this phase, the goshawk will randomly attack the prey. This phase is the global search phase. The mathematical description is given in Eqs (12)–(14):

$$P_{i}=X_{k},i=1,2...,N,k=1,2,...,i-1,i+1,...,N$$ (12)

$$x_{i,j}^{new,p1}= \{\begin{array}{c} x_{i,j}+r(p_{i,j}-Ix_{i,j})Fp_{i}<Fi\\ x_{i,j}+r(x_{i,j}-p_{i,j})Fp_{i}\geq Fi \end{array}$$ (13)

$$X_{i}= \{\begin{array}{c} X_{i}^{new,p1}F_{i}^{new,p1}<F_{i}\\ X_{i}F_{i}^{new,p1}\geq F_{i} \end{array}$$ (14)

where *P*_*i*_ is the orientation of the *i*th northern goshawk that selects its prey, *F*_*pi*_ is the value of its objective function, which also represents the value of fitness, *k* is a random natural number in the interval [1, *N*], $X_{i}^{new,p1}$ is the updated position of the *i*th northern goshawk in the northern goshawk population, $X_{i,j}^{new,p1}$ is the value of its *j*th dimension, $F_{i}^{new,p1}$ is the corresponding fitness, *r* is a random number in the interval [0, 1], and *I* is valued at either 1 or 2. The parameters *r* and *I* are used as random numbers for the search and update behaviour of the northern goshawk in the search.

(3) Phase 2: development phase. After attacking the prey in the previous stage, the prey will escape, and the goshawk will continue to follow and complete the hunt. This stage is the local search of the search space of the algorithm [32]. In the NGO algorithm, the attack radius of this hunt is assumed to be *R*. In the second stage, Eqs. (15)–(17) are used for mathematical modeling:

$$x_{i,j}^{new,p2}=x_{i,j}+R(2r-1)x_{i,j}$$ (15)

$$R=0.02(1-\frac{t}{T})$$ (16)

$$X_{i}= \{\begin{array}{c} X_{i}^{new,p2}F_{i}^{new,p2}<F_{i}\\ X_{i}F_{i}^{new,p2}\geq F_{i} \end{array}$$ (17)

where *t* is the current iteration number of the northern goshawk, *T* is the maximum iteration number, $X_{i}^{new,p2}$ is the new position of the ith goshawk in the second stage, $X_{i,j}^{new,p2}$ is its value of the jth dimension, and $F_{i}^{new,p2}$ is the corresponding fitness of the NGO in the second stage.

### Hybrid improvede Northern Goshawk optimization algorithm

Although the NGO algorithm performs well in terms of convergence accuracy and stability, it still has some limitations:

1) In the process of initializing the population, randomly distributed and non-uniform initial solutions are generated, leading to a decrease in the diversity of the initial population and preventing the algorithm from finding the optimal solution.
2) In the search phase, each dimension of the northern goshawk is reduced, gradually narrowing the search space and increasing the probability of the algorithm falling into a local optimum.
3) The search speed of the algorithm is too fast in the later stage, increasing the likelihood of the algorithm falling into the local optimum.

To solve the above problems, this paper adopts refraction opposition-based learning, sine cosine strategy, and *Lévy* flight strategy to improve the NGO algorithm.

#### Refract opposition-based learning.

**Fig 3:**
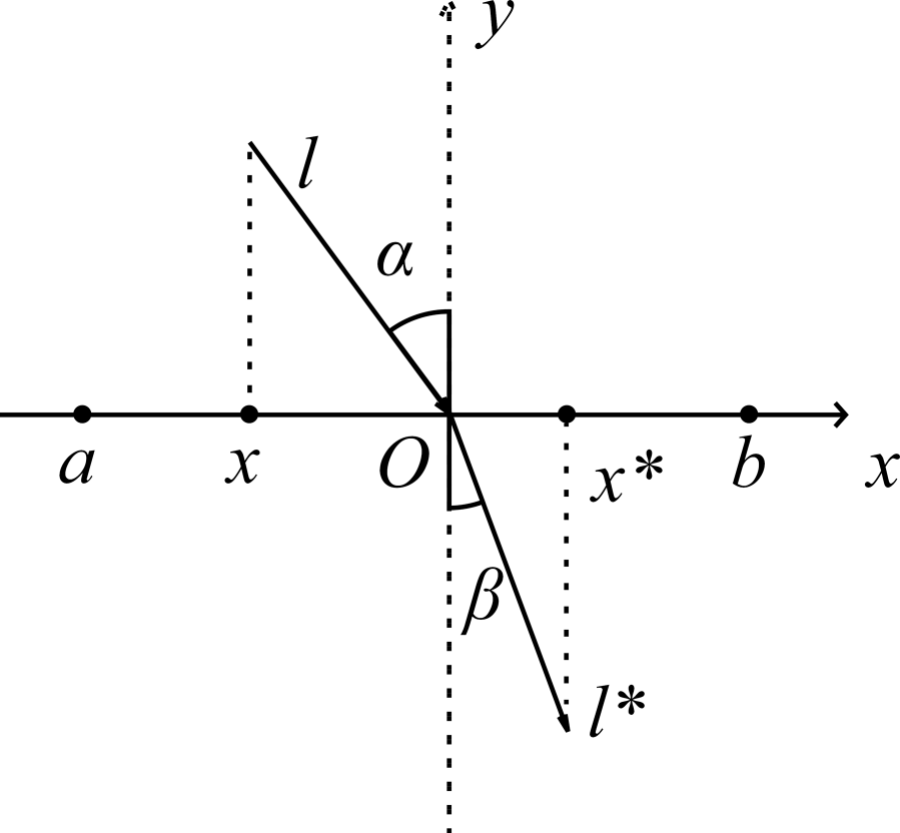
The refracted opposition-based learning mechanism.

Refracted Opposition-Based Learning (ROBL) combines opposition-based learning and the law of light refraction to find better candidate solutions [33]. The refraction opposition-based learning mechanism is introduced in the process of population individual initialization to simulate the refraction process of light to improve the diversity of the population [34]. ROBL enhances the diversity of newly generated solutions by calculating opposite solutions based on randomly generated positions. This approach allows new positions to be more flexible and effectively shifts from the original random locations, guiding the search towards potentially high-quality areas. At the same time, ROBL considers the current solution and its opposite solution to generate better candidate solutions based on fitness values. This enables the early selection of positions with better fitness. This method improves the quality of the initial population, enhancing the overall performance of the algorithm. Fig 3 shows the basic principle of ROBL.

In the Fig 3 The search interval is [*a*, *b*], the *y*-axis represents the normal line, the incident ray length *l*, the refracted ray l*, *α*, and *β* are the shooting Angle and refraction Angle respectively, and the intersection point *O* is the midpoint of [*a*, *b*]. The figure shows that:


sinα=((a+b)/2−x)/l (18)

$$\sin\beta =(x^{*}-(a+b)/2)/l^{*}$$ (19)

From the refractive index definition *n* = sin *α /* sin *β*, combined with Eqs. (18) and (19), the following is obtained.


$$n=\frac{l^{*}((a+b)/2-x)}{l(x^{*}-(a+b)/2)}$$ (20)

The Setting $k=l/l^{*}$, we get:


$$kn=\frac{\left((a+b)/2-x\right)}{\left(x^{*}-(a+b)/2\right)}$$ (21)

After the transformation, the calculation formula of the refraction opposition-based learning solution is obtained as:


$$x^{*}=\frac{a+b}{2}+\frac{a+b}{2kn}-\frac{x}{kn}$$ (22)

When the spatial dimension increases, the formula for the refracted opposition-based learning solution is as follows.


$$x_{i,j}^{*}=\frac{a_{j}+b_{j}}{2}+\frac{a_{j}+b_{j}}{2k}-\frac{x_{i,j}}{k}$$ (23)

where, $x_{i,j}$ is the position of the ith individual in the current population on the jth dimension, $x_{i,j}^{*}$ is the inverted refraction solution of $x_{i,j}$, $a_{j}$ and $b_{j}$ are the minimum and maximum values of the *j*th dimension in the search space.

**Fig 4:**
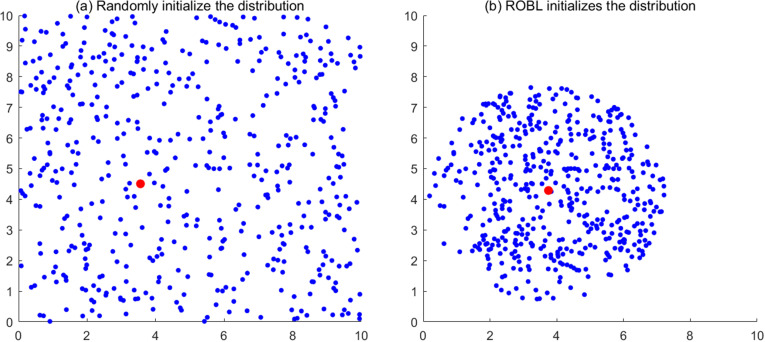
Comparison of population distribution before and after improvement.

Fig 4 compares scatter plots of populations initialized with random methods and those initialized with ROBL, with red points representing individuals with optimal fitness. The figure shows a clear change in population distribution before and after optimization using Refracted Opposition-Based Learning (ROBL). After optimization, the individuals are distributed more directionally toward the optimal solution area.

#### The sine-cosine strategy.

The sine-cosine algorithm possesses oscillatory characteristics based on the sine and cosine functions, which can enhance the global search ability and prevent the algorithm from falling into local optimal solutions [35]. To address the phenomenon of local optimality in the exploration phase, the sine-cosine algorithm replaces the position update formula of the original Northern Goshawk algorithm.

The basic step size search factor of the sine cosine algorithm is as follows.


$$r_{1}=1-\frac{t}{Iter_{max}}$$ (24)

where *t* is the current iteration number. Since this search factor shows a linear decreasing trend, it cannot effectively balance the global search and local development capabilities of NGO algorithms. Nonlinear decreasing search factor is adopted as in Eq. (25):


$$r2={\left[1-{\left(\frac{t}{Iter_{\max}}\right)}^{\eta }\right]}^{1/\eta }$$ (25)

where By adjusting the coefficient *η* (*η* >1) and the initial value, the local development ability of the Northern Goshawk algorithm was enhanced.

The nonlinear weight factor of Eq. (26) is introduced to reduce the dependence of the individual position update of the goshawk on the current position.


$$\omega =\frac{e^{\frac{t}{\text{Max\_iter}}}-1}{e-1}$$ (26)

where: *t* is the current iteration number, Max_iter is the maximum iteration number, and *e* is the base of the natural logarithm. The weight factor increased with the number of iterations, and the global optimization ability of the algorithm was improved in the early stage. The convergence speed of the algorithm is accelerated in the later stage. Finally, the goshawk position update formula is obtained as Eq. (27).


$$X_{i,j}^{t+1}= \{\begin{array}{c} \omega \cdot X_{i,j}^{t}+r_{2}\cdot \sin r_{3}\cdot |r_{4}\cdot X_{best}-X_{i,j}^{t}|R_{2}<ST\\ \omega \cdot X_{i,j}^{t}+r_{2}\cdot \cos r_{3}\cdot |r_{4}\cdot X_{best}-X_{i,j}^{t}|R_{2}\geq ST \end{array}$$ (27)

where *r*_3_∈[0, 2π] and *r*_4_∈[0, 2].

#### Lévy flight strategy.

*Lévy* flight strategy is a probability distribution model proposed by mathematician *Lévy*, following *Lévy* distribution and performing random search operations [36]. In this study, we adopt the *Lévy* flight strategy to refine and enhance the current optimal solution of the algorithm. *Lévy* flight is calculated as follows.


$$\text{L}={\frac{\mu }{\left|v\right|}}^{-\beta }$$ (28)

where: *β*∈(0, 2); *µ* and *v* are direction vectors that follow *N* (0, *σ*^2^) and *N* (0, 1) distributions, respectively. Where: Γ(x)=(x−1)!.


$$\sigma ={\left\{\frac{\Gamma (1+\beta )\sin(\pi \beta /2))}{\Gamma (1+\beta )/2]\beta \cdot 2\left[\left(\beta -1\right)/2\right]}\right\}}^{1/\beta }$$ (29)

In the second stage of NGO, the position of goshawk is updated using the *Lévy* flight strategy as follows.


$$X_{i}= \{\begin{array}{c} x_{i,j}+R(2q-1)x_{i,j}\cdot Levy(\beta )F_{i}^{new,p_{2}}<F\\ x_{i,j}F_{i}^{new,p_{2}}\geq F \end{array}$$ (30)

#### The AdaBoost algorithm.

**Fig 5:**
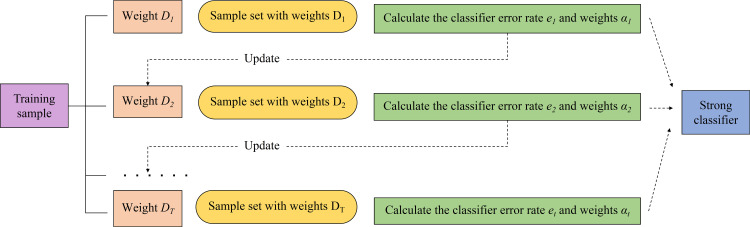
AdaBoost training flowchart.

The main principle of AdaBoost is to apply different sample weights to the same training set, then bring them into multiple weak classifiers, and combine the diagnostic results into a strong classifier by weighting [37]. The AdaBoost training flow is shown in Fig 5.

The specific steps of the algorithm are as follows.

1) Set the initial weights of weak classifiers as follows.

$$D_{1}\left(\text{i}\right)=\frac{1}{N}$$ (31)

where, *D*_1_(*i*) represents the weight of the *i*th sample in the first iteration, and *N* is the number of samples involved in the training of the weak classifier.

2) Set the number of iterations *t*, select the weak classifier *F* with the smallest error rate for iteration, and calculate the error rate of the weak classifier:

$$e_{t}=\sum_{i=1}^{m}D_{i}(i)(F_{t}(x_{i})\neq y_{i})$$ (32)

where, *e*_*t*_ is the error rate, *F*_*t*_ (*x*_*i*_) is the predicted label, which represents the predicted label of *F* for the *i*th sample. *y*_*i*_∈{-1, 1} is the actual label of the *i*th sample, and set -1 to be the wrong label and 1 to be the correct label.

3) Calculate the weights of the weak classifiers:

$$\alpha_{t}=\frac{1}{2}\ln\frac{1-e_{t}}{e_{t}}$$ (33)

4) Update the training set weights:

$$D_{t+1}(i)=\frac{D_{t}(i)\exp(-\alpha_{t}y_{i}F_{t}(x_{i}))}{2\sqrt{e_{t}(1-e_{t})}}$$ (34)

where, when the sample is correctly classified, *y*_*i*_*F*_*t*_(*x*_*i*_)=1, and the classification error *y*_*i*_*F*_*t*_(*x*_*i*_)=-1. The sample weight update formula is as follows:


$$D_{t+1}{\left(i\right)}^{+}=\frac{D_{t}(i)}{2(1-e_{t})}$$ (35)

$$D_{t+1}{\left(i\right)}^{-}=\frac{D_{t}(i)}{2e_{t}}$$ (36)

where, *D*_*t*+1_(*i*)^+^ represents the weight of classified correct samples, and *D*_*t*+1_(*i*)^-^ represents the weight of classified correct samples.

5) Repeat the above steps until the maximum number of iterations *T* is reached, and finally synthesize a strong classifier according to the weight of the weak classifier:

$$F_{final}=sign(\sum_{t=1}^{T}\alpha_{t}h_{t}(x))$$ (37)

*F*_*final*_ is the final model, and the AdaBoost algorithm is trained through continuous iteration. When some samples are misclassified multiple times, the weights of such samples will increase, leading to a degradation in the model’s performance. To inhibit the excessive growth of the weights of misclassified samples, this paper proposes an Improved AdaBoost algorithm (IAdaBoost) that incorporates the misclassification frequency of samples into the weight calculation formula. The updated weight calculation formula is as follows: Eq. (38):


$$D_{t+1}(i)= \{\begin{array}{c} \frac{D_{t}(i)}{2(1-e_{t})}\\ {\left(\frac{D_{t}(i)}{2e_{t}}\right)}^{\frac{1}{n}} \end{array}$$ (38)

*D*_*t*+1_(*i*) represents the improved weight iteration formula of misclassified samples, and *n* is the number of misclassified samples. By inhibiting weight growth, the classification errors during the algorithm’s training process were reduced, allowing the algorithm to focus more on optimizing global data and avoiding a decline in overall accuracy due to a few misclassified samples.

### HINGO-LSSVM-IAdaBoost fault diagnosis model

**Fig 6:**
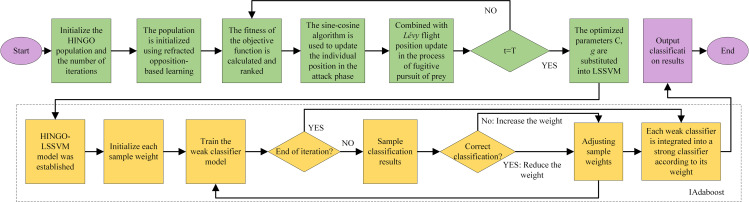
Flow chart of the HINGO-LSSVM-IAdaBoost algorithm.

The HINGO algorithm is used to optimize the penalty factor *C* and kernel parameter *g* of LSSVM, and IAdaBoost is used as the framework to further enhance the diagnosis algorithm adaptively to improve the fault classification effect of the model. Fig 6 shows the flow chart of the HINGO-LSSVM-IAdaBoost algorithm.

Step 1: Set the population number and iteration number; The fitness of the objective function was calculated and sorted, and the current optimal solution and fitness value were saved.

Step 2: After randomly initializing the population of goshawk, the population position is optimized by refraction opposition-based learning.

Step 3: Replace the original northern goshawk formula with the sine cosine algorithm to update the position of the ith northern goshawk in the *j*the dimension in the first stage. In the second stage, the position is updated by combining *Lévy* flight strategy.

Step 4: Check whether the algorithm satisfies the maximum number of iterations. If so, derive the optimal solution obtained by HINGO; The optimized parameters *C* and *g* were substituted into LSSVM.

Step 5: Establish the current optimal HINGO-LSSVM weak classifier, initialize the weight of each sample.

Step 6: Train the weak classifier model, calculate the error, and update the weight according to the classification results.

Step 7: After the iteration, integrate the strong classifier according to the weight of each weak classifier.

Step 8: Output the final HINGO-LSSVM-IAdaBoost fault diagnosis model of the chiller unit.

## Chiller fault units diagnosis experiment

### Model performance evaluation metrics

This paper employs a multi-classification evaluation framework based on the confusion matrix and three sets of evaluation metrics to rigorously assess the performance of the enhanced chiller fault diagnosis model.

(1) The multiclassification confusion matrix is shown in Table 2. Based on this confusion matrix, the accuracy rate (*Acc*), individual category fault diagnosis accuracy rate (*AR*), underreporting rate (*UR*), and false alarm rate (*FAR*) are defined.

**Table 2:** Confusion matrix diagram.

Category		Prediction class		
		1	2	3
**Real classes**	1	*a*	*b*	*c*
	2	*d*	*e*	*f*
	3	*g*	*h*	*i*

*Acc* is defined as follows:


Acc=(a+e+i)/(a+b+c+d+e+f+g+h+i) (39)

Taking Category 1 as an example, the definitions of *AR*, *UR*, and *FAR* are shown in Eqs. (40)–(42):


AR=a/(a+b+c) (40)

UR=(b+c)/(a+b+c) (41)

FAR=(d+g)/(a+d+g) (42)

(2) Precision (*P*) is used to evaluate the accuracy of the model in diagnosing a specific fault class. Recall (*R*) evaluates the model’s ability to detect a specific fault class. The *F*1 score is the reconciled average of precision and recall, providing a comprehensive evaluation of the model’s performance. The higher the *F*1 score is, the better the model balances between precision and recall.

$$P=\frac{TP}{TP+FP}$$ (43)

$$R=\frac{TP}{TP+FN}$$ (44)

Among them, *TP* stands for true positive, *TN* for true negative, *FP* for false positive and *FN* for false negative. It is obtained according to Eqs. (43)–(44):


$$F1=\frac{2PR}{PR}$$ (45)

(3) The *Kappa* coefficient(*Kappa*) reflects the consistency between real classification and predicted classification.

$$Kappa=\frac{p_{0}-p_{e}}{1-p_{e}}$$ (46)

Among them, *p*_0_ is the number of correctly predicted samples divided by the total number of samples. Assuming that the true samples for each class are *a*_1_, *a*_2_, …, *a*_e_, and the predicted unclassified samples are *b*_1_, *b*_2_, …, *b*_e_, respectively.


$$p_{e}=\frac{a_{1}b_{1}+a_{2}b_{2}+\cdots +a_{n}b_{n}}{n^{2}}$$ (47)

The *Kappa* coefficient values range from [0, 1], and the closer the *Kappa* coefficient is to 1, the better the model’s diagnostic performance.

## Results and analysis

To select the classifier with the best early fault diagnosis effect for chillers, this paper selects seven benchmark models for comparison, and evaluates them by *Acc*, *F*1 score and *Kappa* coefficient.

**Fig 7:**
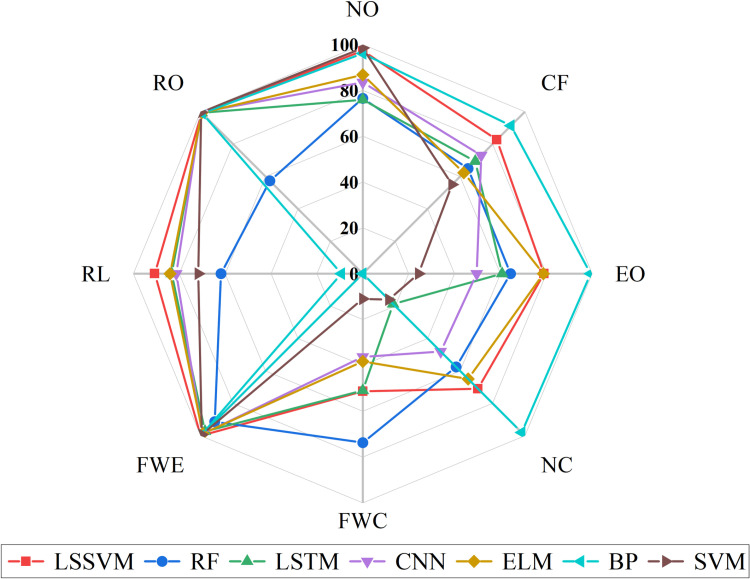
*AR* of the seven benchmark fault diagnosis model.

Seven benchmark fault diagnosis models were analyzed using the RP-1043 dataset, with results shown in Fig 7 and Table 3. The diagnostic accuracy was generally low, with only the BP neural network and LSSVM achieving over 80% accuracy. As seen in Fig 7, the BP neural network showed notably low accuracy for some faults, leading to invalid *F*1 score calculations (NaN) and unstable performance. Thus, LSSVM was identified as the most effective classifier.

**Table 3:** Fault diagnosis results of seven benchmark models.

Fault diagnosis method	SVM	RF	LSTM	CNN	ELM	BP	LSSVM
***F*1**	0.6338	0.6746	0.7022	0.7432	0.7903	NaN	0.8361
***Kappa***	0.6750	0.6676	0.6769	0.7165	0.7694	0.7770	0.8258
***Acc*(%)**	70.09	70.67	71.21	74.64	79.42	80.12	84.36

The results shown in Fig 7 can be analyzed as follows. The differences in feature extraction capabilities of different models affect the fault diagnosis results. SVM, as a binary classification model, is sensitive to the class boundary and distribution, performing poorly when feature similarity is high, especially in diagnosing EO, NC, and FWC faults, with lower accuracy. RF, based on decision tree ensemble, can effectively handle nonlinear relationships and resist noise, but it may have significant errors in complex nonlinear problems, especially in diagnosing NO, CF, and RO faults. LSTM is suitable for processing sequential data, but due to the weak temporal correlation in chiller fault data, it performs poorly, especially in non-sequential faults like EO and NC. CNN is good at image recognition but performs worse than other models in feature extraction for multidimensional data and fault diagnosis, leading to lower accuracy. ELM, while fast in training and strong in generalization, has low accuracy when dealing with hard-to-distinguish categories like CF and FWC. BP network has strong nonlinear fitting ability but is prone to getting stuck in local minima and requires high data preprocessing. It performs well in diagnosing EO and NC faults but poorly in diagnosing FWC and RL categories. LSSVM has strong generalization ability in handling high-dimensional feature data, especially in diagnosing NO and RO categories, but is not as effective in diagnosing complex or similar categories like FWC, EO, and NC. In addition, the fault categories themselves have different characteristics. NO and RO faults have more distinct features and are easier to distinguish, so models perform better in recognizing these categories. However, faults like FWC, EO, and NC have more similar features, making diagnosis more challenging.

To validate the proposed optimization algorithm’s effectiveness in improving chiller units fault diagnosis accuracy, this study uses the HINGO algorithm along with classical algorithms like PSO, WOA, and SSA to optimize the LSSVM model and establish a fault diagnosis framework, utilizing the RP-1043 dataset for experiments. The population size for all algorithms is set to 30, and the number of iterations is set to 15 to ensure consistency. The impact of different optimization algorithms on diagnosis accuracy is analyzed, with results shown in Table 4.

**Table 4:** Model comparison analysis results of LSSVM with different optimization algorithms.

Fault diagnosis method	*R*	*P*	*F*1	*Kappa*	*Acc(%)*
**LSSVM**	0.7941	0.9277	0.8361	0.8258	84.36
**PSO-LSSVM**	0.8676	0.9422	0.8979	0.8810	89.39
**WOA-LSSVM**	0.8765	0.9427	0.9020	0.8873	89.97
**SSA-LSSVM**	0.8735	0.9509	0.9036	0.8902	90.21
**NGO-LSSVM**	0.9347	0.9608	0.9466	0.9344	94.18
**KPCA-LSSVM-GSA [** **23** **]**	—	—	—	—	95.70
**HINGO-LSSVM**	0.9609	0.9729	0.9660	0.9589	96.36

As shown in Table 4, the NGO-LSSVM fault diagnosis model achieves an accuracy of 94.18%, exceeding the PSO, WOA, and SSA algorithms, highlighting the strong optimization capability of the NGO algorithm. After LSSVM parameter optimization using the HINGO algorithm, the model’s accuracy reaches 96.36%, a 2.18% improvement over the NGO-LSSVM model. This indicates that the improvement strategy effectively enhances optimization performance and diagnostic accuracy. Compared to the early fault diagnosis model for chilled water units based on KPCA-LSSVM-GSA proposed in literature [23], the accuracy improved by 0.66%.

The introduction of a refractive reverse learning strategy during the initialization phase increases population diversity, allowing for a more rational distribution of the population in the search space. This avoids uneven population distribution caused by randomly generating the initial population in the standard northern goshawk optimization algorithm. It indirectly expands the search range and optimizes the values of the initial positions. This enhances the algorithm’s convergence performance. In the development phase, an improved sine-cosine strategy replaces the standard northern goshawk position update strategy. The periodicity and oscillation of the sine-cosine function enable updated northern goshawk individuals to obtain stronger exploratory capabilities, enhancing both local and global search abilities. Additionally, the introduction of a nonlinear convergence factor and *Lévy* flight strategy further improves the algorithm’s local optimization capabilities in later stages, allowing the proposed HINGO to effectively explore the search space and ensure strong global and local search capabilities.

**Fig 8:**
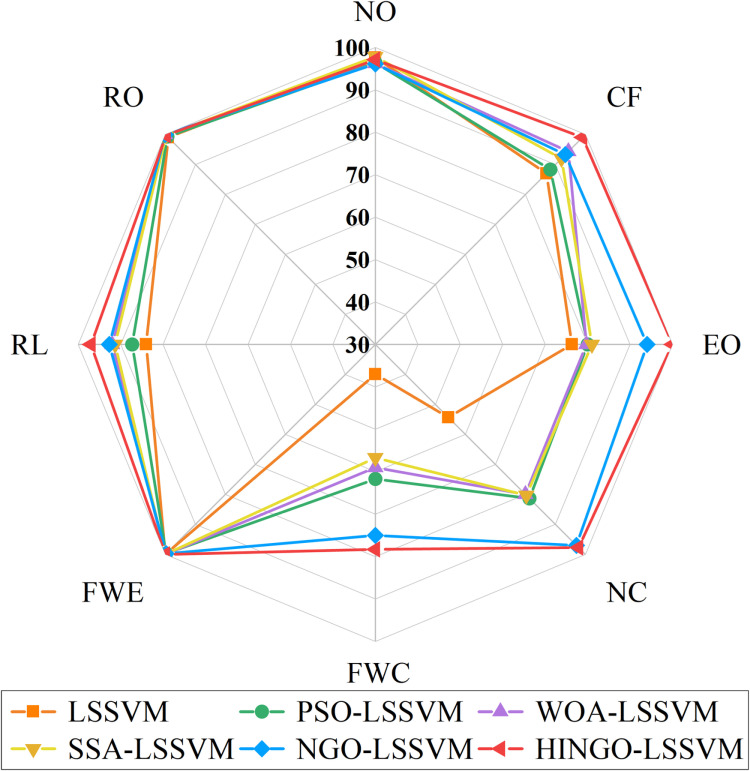
*AR* of LSSVM with different optimization algorithms.

Fig 8 shows the diagnostic results of the LSSVM model under different optimization algorithms for various faults. For faults such as NO, CF, EO, and RO, these faults are more obvious and their features are more distinct, resulting in higher diagnostic accuracy with little difference between the optimization algorithms. For faults such as FWC, FWE, RL, and NC, due to their complex nonlinear features and hidden symptoms, the LSSVM model struggles to extract key features, leading to poorer discrimination capability. In terms of optimization algorithms, the PSO algorithm has strong global search ability and can effectively adjust the LSSVM parameters, but its local search ability is weak and prone to getting stuck in local optima, resulting in poor performance for fault categories such as NC and FWC. The WOA algorithm, when handling complex nonlinear problems, avoids local optima better, thus achieving higher diagnostic accuracy for categories such as CF and RL. The SSA algorithm has strong local search ability but lacks precision in classifying complex samples, leading to higher accuracy for NO and CF, but poor performance for FWC. The NGO algorithm performs exceptionally in dealing with nonlinear classification problems, significantly improving overall accuracy, particularly for the classification of EO, NC, and FWC faults. The HINGO algorithm further enhances computational efficiency and optimization ability, performing excellently in categories such as EO, NC, and FWE, showing its comprehensive advantages in multi-fault type classification.

**Fig 9:**
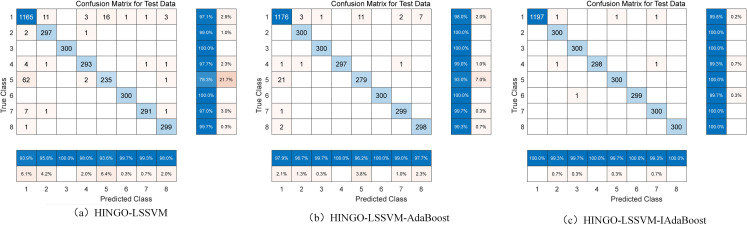
Confusion matrix of model diagnosis result.

Metrics such as *R*, *P*, *F*1 score, and *Kappa* coefficient indicate that the HINGO-LSSVM model outperforms other control groups, and this model achieves the highest accuracy for each fault type. Overall, the HINGO-LSSVM model demonstrates superior diagnostic capabilities compared to other models, confirming that the proposed HINGO optimization algorithm outperforms other algorithms. The confusion matrix for the HINGO-LSSVM model is presented in Fig 9a.

To further enhance fault diagnosis performance, the HINGO-LSSVM model is integrated with the IAdaBoost algorithm, resulting in the HINGO-LSSVM-IAdaBoost adaptive fault diagnosis model. A control group using the HINGO-LSSVM-AdaBoost model was established to validate the improved IAdaBoost algorithm. The fault diagnosis confusion matrices are presented in Fig 9b and 9c. At the same time, To evaluate the effectiveness of each enhancement module in the proposed fault diagnosis model, the five models LSSVM, NGO-LSSVM, HINGO-LSSVM, HINGO-LSSVM-Adaboost and Hingo-LSSVM-IAdaboost are compared and analyzed. Table 5 lists the fault diagnosis results for each evaluation metric, where bold values indicate superior performance.

**Table 5:** Model comparison analysis results.

Assessment of indicators	*R*	*P*	*F*1	*Kappa*	*Acc*(%)
**LSSVM**	0.7941	0.9277	0.8361	0.8258	84.36
**NGO-LSSVM**	0.9347	0.9608	0.9466	0.9344	94.18
**HINGO-LSSVM**	0.9609	0.9729	0.9660	0.9589	96.36
**HINGO-LSSVM-AdaBoost**	0.9862	0.9865	0.9863	0.9825	98.45
**HINGO-LSSVM-IAdaBoost**	**0.9984**	**0.9975**	**0.9980**	**0.9979**	**99.82**

The HINGO-LSSVM model served as the weak classifier, and the improved IAdaBoost algorithm increased diagnostic *Acc* by 1.37% compared to the traditional AdaBoost. Table 5 shows that the *F*1 for the HINGO-LSSVM-IAdaBoost model is 0.9980, close to the ideal value of 1, indicating a good balance between recall and precision. Additionally, the *Kappa* coefficient is 0.9979, demonstrating high consistency between predicted results and actual labels.

The fault diagnosis results of the five models, LSSVM, NGO-LSSVM, HINGO-LSSVM, HINGO-LSSVM-adaboost, and HINGO-LSSVM-iadaboost, in Table 5 show significant improvements in *Acc*, *R*, *P*, *F*1, and *Kappa*. The LSSVM model, as the base model, demonstrates good performance. However, its accuracy is highly sensitive to the model’s key parameters when dealing with complex data structures, leading to potential overfitting issues. To address this, the NGO algorithm was used to optimize the parameters of the LSSVM, enhancing the model’s robustness and adaptability, enabling better performance in more complex scenarios. Building on this, the HINGO algorithm was further employed to reduce overfitting risk and improve the model’s generalization ability. The HINGO-LSSVM model effectively improves fault diagnosis accuracy, especially under high noise and complexity in the data, showing more robust performance than the traditional LSSVM model. To further enhance the model’s ability to handle difficult-to-classify samples, the AdaBoost algorithm was introduced, improving the diagnosis of challenging samples by combining the predictions of multiple classifiers. However, the traditional AdaBoost algorithm is overly sensitive to misclassified samples, which can lead to overfitting on noisy data. Therefore, this study proposes the IAdaBoost algorithm, which optimizes the weight update rule, balancing sensitivity to both misclassified and correctly classified samples, thereby effectively improving the fault diagnosis model’s performance in various scenarios. The significant improvements of the five models across multiple evaluation metrics validate the importance of each improvement module in enhancing fault diagnosis accuracy and robustness. These improvements enable the model to better cope with complex and uncertain environments in practical applications, enhancing fault diagnosis efficiency and reliability.

**Fig 10:**
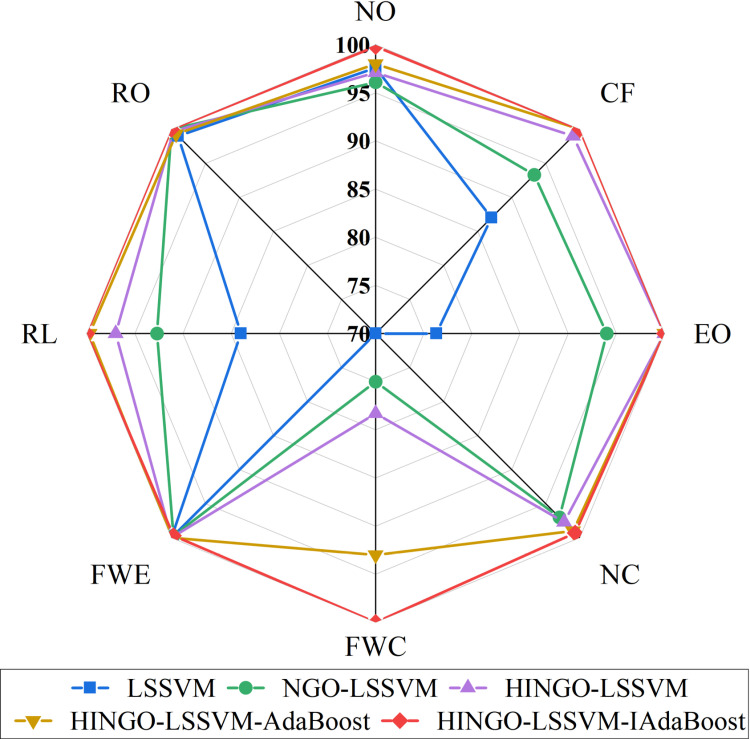
*AR* of the five fault diagnosis model.

Fig 10 highlights that this model achieves the highest individual category fault diagnosis accuracy rate (*AR*). The LSSVM model is relatively simple and struggles with diagnosing complex faults, especially in feature extraction and handling nonlinear relationships. Its fault diagnosis accuracy is also highly influenced by hyperparameters. After introducing the NGO optimization algorithm, its overall performance is significantly better than the standard LSSVM. NGO better adjusts model parameters, reduces overfitting, and improves classification accuracy. It is particularly stable when handling complex fault categories like CF, EO, and NC. However, the improvement in identifying FWC faults is still not significant. The HINGO-LSSVM model improves performance further through enhancements to the optimization algorithm, showing considerable progress in diagnostic accuracy, especially for CF, EO, and FWE categories. For FWC, although accuracy has improved, there is still room for further improvement. By combining the AdaBoost ensemble learning method and training multiple HINGO-LSSVM weak classifiers, the model’s robustness and stability are enhanced. At the same time, AdaBoost improves the ability to identify the harder-to-diagnose FWC category. Although the accuracy is still lower than for other categories, it has improved compared to HINGO-LSSVM. The HINGO-LSSVM-IAdaBoost model optimizes the weight update rules based on the AdaBoost strategy, allowing better adjustment of the model’s handling of difficult-to-classify categories. This results in a significant enhancement in the model’s ability to classify the FWC category.

## Conclusion

Traditional fault diagnosis methods for chillers lack accuracy and stability, and early operational fault diagnosis research has been insufficient. This study proposes an HINGO-LSSVM-IAdaBoost method for early fault diagnosis in chillers. Key findings are summarized below.

This paper proposes a diagnostic model for early fault detection in chiller units. First, the HINGO algorithm, based on the NGO algorithm, enhances solution accuracy and convergence speed by improving population initialization, balancing global and local search with a sine-cosine strategy, and using *Lévy* flight to avoid local optima. In fault diagnosis, HINGO optimizes LSSVM, achieving higher accuracy than other optimization algorithms. Next, the traditional AdaBoost algorithm is improved to propose the IAdaBoost algorithm, and the HINGO-LSSVM model is subjected to ensemble learning. The HINGO-LSSVM-IAdaBoost model achieves 99.84% accuracy, outperforming other models in various evaluation metrics, confirming the effectiveness of the proposed improvements, and proving that the model has high reliability and accuracy for early fault detection in chillerr units. Ablation experiments validate the necessity and effectiveness of each improvement strategy.

In summary, the fault diagnosis model based on HINGO-LSSVM-IAdaBoost proposed in this paper can accurately identify early fault types in chiller systems. This provides an effective and reliable method for fault diagnosis in this field.

However, this study did not validate the theoretical model using actual operational data from the chiller system. Due to the complexity of real-world data, this may lead to discrepancies between the theoretical model and actual signals. In the future, we plan to apply the model to chiller units fault detection to further verify its effectiveness. Another important aspect of air conditioner condition monitoring is predicting the remaining useful life. In future research, we expect that combining the HINGO algorithm with other advanced prediction techniques will offer a more comprehensive solution for chiller operation management.
